# Integrated assessment of water security in D-8 countries

**DOI:** 10.1016/j.heliyon.2024.e39781

**Published:** 2024-10-24

**Authors:** Azam Bahramifard, Mansour Zibaei

**Affiliations:** Department of Agricultural Economics, College of Agriculture, Shiraz University, Shiraz, Iran

**Keywords:** Water security, Data envelopment analysis model, D-8 countries

## Abstract

In terms of evaluating the water security situation of communities, it is necessary to use appropriate indicators. To do this, composite indicators are especially useful for policy-making on water resources, aiming to enhance water security, implementation, and monitoring of policy actions. The composite indicators of water security at the national level reflect the country's status in terms of water resource sustainability, human well-being, and water environment. In this study, the composite indicators of water security for D-8 countries were investigated in various dimensions using the data envelopment analysis model. Moreover, the status of selected countries was examined with regard to indicators related to the dimension of water security sustainability. The results provided by the proposed model showed that Malaysia, Bangladesh, Indonesia, Nigeria, and Turkey have better positions regarding the sustainability dimension compared to other countries. The water exploitation intensity in these countries was less than 40 %. Furthermore, the level of water availability in Malaysia, Bangladesh, and Indonesia was higher than 7000 cubic meters per person. It is worth noting that Low exploitation intensity and high availability of water in a country increased its water security in terms of sustainability dimension. The low water availability and very high water exploitation in Iran, Pakistan, and Egypt exacerbate their insecurity in the sustainability dimension. Due to the importance of the sustainability dimension in determining the water security status of countries, it is recommended to take this dimension into account.

## Highlights

• The data envelopment analysis model is used to create composite indicators.
• Water security is evaluated separately in each security dimension.
• Water security dimensions can be used as the basis for countries comparison.
• The sustainability dimension has an influential role in improving water security.

## 1 Introduction

### 1.1 Background

Water is one of the main elements of sustainable development which is necessary for various activities such as agriculture, energy production, exploitation of resources, industrial growth, business activities, protection of the ecosystem, and a variety of other utilizes [1]. Moreover, this vital and relatively scarce resource is particularly effective in the establishment and preservation of human security in relation to the environment [2]. In addition, it influences the food security of regions and national security [3,4]. Water has also become a sensitive and political topic due to water pollution, overexploitation of groundwater resources, and lack of necessary infrastructure [2].

Due to the rapid growth of population, expansion of urbanization, migration, and changing consumption patterns, the demand for water resources has enhanced [5,6], so an increase in demand and competition in various sectors for water resources put pressure on water resources [5]. The decline in food production resulting from water scarcity and a growing population leads to worsened living conditions, environmental problems, and challenges of water resource limitations. Consequently, these issues cause poverty, malnutrition, and famines. If countries are unable to change their irrigation and agricultural practices, the reduction of food production, arable land, and water in these countries will increase their dependence on other countries to meet their food needs [2]. Furthermore, changes in the hydrological cycle caused by human activities also affect the water cycle and availability [5]. Climate change can be considered as the main factors that lead to changes in the water cycle, hydrological cycle, increase in the possibility of severe droughts and devastating floods. These may exert significant effects on the water security of communities [5,6]. Land use changes and deforestation also influence the hydrological and water cycle [5]. The water management has a great effect on the presence or absence of water-related problems. In one case, although the natural conditions of a region may be completely favorable, the risks of drought, floods, and water pollution can increase due to water dismanagement. In another case, natural conditions may give rise to problems such as water scarcity and floods, while adequate management can reduce these risks [7]. Therefore, all components influencing water resources can raise the dangers and vulnerabilities related to people's security [5].

### 1.2 Literature review

In recent years, numerous countries have faced water scarcity, water pollution, and water-related damages [8,9]. With increasing concerns for water resources, politicians, organizations, and investors have used the term “water security" (WS) in order to declare their opinions [3]. This term has attracted much attention from politicians, governmental institutions, and scientific societies [[10], [11], [12]]. Human society has encountered many issues in the last century, but the most significant issue is WS [9]. Since its appearance in the 1990s, WS has undergone substantial development [11]. An integrative definition of WS was provided by the Global Water Partnership (GWP) in 2000. It states “water security, at any level from the household to the global, means that every person has access to enough safe water at affordable cost to lead a clean, healthy, and productive life, while ensuring that the natural environment is protected and enhanced” [3]. The Global Water Partnership has identified WS as the main goal of water resources management [7]. All the principles of integrated water resources management (IWRM), especially the integration principle, are hidden in WS, which leads to the alignment of integrated management and WS [3]. Grey and Sadoff [13] proposed another definition of WS, stating that WS is “the availability of an acceptable quantity and quality of water for health, livelihoods, ecosystems, and production, coupled with an acceptable level of water-related risks to people, environments and economies”.

WS is applicable at various scales, from local to global although each scale should focus on specific issues. Furthermore, it is important to assess the relationship between WS and other security issues, and its significance varies depending on the scale. For example, these relationships are not often identifiable at the basin level and in specific regions [3] while WS impacts food security, energy security, national security, climate security, and human and community security at the national level [3,14]. Therefore, these dependencies should be considered.

WS, as a significant political challenge, requires striking a balance between environmental and human water demands while preserving ecosystems and biodiversity [10,15]. Appropriate indicators are required to address these challenges. These indicators should be effective in motivating policy changes and evaluating the effectiveness of interventions [16]. WS indicators are considered tools for evaluating the results of integrated water resources management. These suggest a fresh perspective to identify the weaknesses and strengths of current water resource management and providing services. WS indicators provide a basis for assessing the results of policies, which can reported to stakeholders. The path and importance of raising investment and enhancing governance in the water sector are also illustrated by these indicators [17]. Policy formulation would be affected by indicators only if indicators are valid [16]. Therefore, suitable indicators help to assess the current status of WS and its changes over time [15,16].

Composite indicators provide comprehensive and more information compared to partial indicators. Besides, these indicators are especially useful for policy-making on water resources, aiming to enhance WS, implementation, and monitoring of policy actions [18,19]. It should be noted that the composite indicator of WS at the national level reflects the country's status in terms of water resource sustainability, human well-being, and water environment [18]. Recently data envelopment analysis (DEA) has gained more popularity as the method for constructing composite indicators [20], which can be an effective and appropriate instrument for enhancing decision-making processes and monitoring water management policies [18]. This approach fails to require expert opinions and judgments, and it performs the weighting and aggregation of a set of partial indicators simultaneously [20].

In recent years, a lot of investigations have been conducted on the concept and assessment of WS, and different articles have used various methods. WS assessment considering the water footprint concept was performed by D'Ambrosio et al. (2020), Veettil and Mishra (2020), Kaur et al. (2019), and Veettil and Mishra (2016) separately. These studies have utilized the water footprint concept to analyze the WS status at the basin level. Investigations were performed by de Castro-Pardo et al. (2022), and Park et al. (2020) at the level of countries. WS has been evaluated in some investigations using different techniques of the multi-attribute decision-making (MADM) method [9,[21], [22], [23], [24], [25], [26], [27], [28], [29]]. The review of the conducted studies shows that the assessment of WS at different levels, from the household level to the global level, has attracted the attention of many researchers. In spite of this popularity, to the best of the authors' knowledge, no studies so far have investigated the status of WS for D-8 countries. The main objective and novelty of this investigation are to assess the situation of WS considering different dimensions in D-8 countries.

The objectives of the present paper are as follows.

• Assessing the WS situation in selected countries using DEA model
• To examine the situation of sustainability indicators in selected countries
• To identify the critical countries in terms of sustainability indicators

Due to the lack of updated data, the latest available data was used. The current investigation shows the WS situation in 2020–2022. This investigation helps to identify countries that have relatively high or low WS in each dimension of WS compared to other countries. It also helps to know the dimensions that increase or decrease the WS of a country compared to other countries. Therefore, knowledge of the weak dimensions of WS in each country, which leads to its lower ranking compared to other countries, is a good guide for suggestions and necessary actions to improve WS in each security dimension.

## 2 Materials and methods

### 2.1 Study area

In this investigation, several developing Islamic countries in Asia and Africa were selected, including Iran, Turkey, Pakistan, Egypt, Nigeria, Bangladesh, Malaysia, and Indonesia. The purpose of forming this group (D-8 countries) was to establish regional agreements for creating strong economic relationships and increased penetration in global markets. The population of D-8 countries is 1.236 billion people, meaning one in every seven people in the world is living in D-8 group. Understanding the WS status of this group is especially important because it is equivalent to the WS status of 15 % of the world's population.

### 2.2 Selection of assessment indicators

**Fig. 1:**
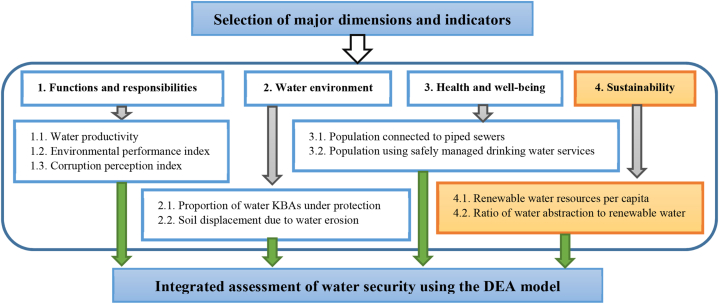
The overview of assessment framework.

Several frameworks have been proposed to assess WS at the national level. Frameworks are usually based on dimensions that enable national comparisons. The selected evaluation framework is shown in Fig. 1. WS consists of four major dimensions: functions and responsibilities of societies, water environment, health and well-being, and sustainability [18,30]. In this investigation, the water productivity, environmental performance index (EPI), and corruption perception index (CPI) were chosen as indicators to reflect the current condition of functions and responsibilities of societies. The proportion of water key biodiversity areas (KBAs) under protection and the soil displacement due to water erosion are applied to assess the water environment. Population connected to piped sewers and population using safely managed drinking water services were selected as the indicators to represent health and well-being. The renewable water resources per capita and the ratio of water abstraction to renewable water were selected as indicators to reflect the current state of sustainability dimension. The renewable water resources per capita, the ratio of the total renewable water resources to the population, was used to indicate the amount of available water. The ratio of water abstraction to renewable water shows the intensity of water exploitation.

The indicators have been collected and calculated using the latest available data. Water withdrawal, water productivity, and countries' populations were collected from the World Bank database. The corruption perception index and environmental performance index were collected from Transparency International and the results of the environmental performance index, respectively. Renewable water and the proportion of the population using safely managed drinking water services were derived from Sustainable Development Goals. The proportion of the population connected to piped sewers was collected from the World Health Organization, and the proportion of water key biodiversity areas under protection was obtained from statistical data on the land portal. Furthermore, estimates of global soil displacement due to water erosion from a research article were used to calculate the soil erosion rate caused by water. The description of dimensions and indicators are given in Table 1.

**Table 1:** Description of dimensions, indicators, units of measurement, years and source.

Dimensions	Indicators	Units	Years	Source
1. Functions and responsibilities	1.1. Water productivity	constant 2015 US$ GDP per cubic meter of total freshwater withdrawal	2020	https://data.worldbank.org
	1.2. Environmental performance index	(score: 0 to 100)	2022	https://epi.yale.edu
	1.3. Corruption perception index	(score: 0 to 100)	2022	https://www.transparency.org/en/cpi/2022
2. Water environment	2.1. Proportion of water KBAs under protection	percent	2020	https://www.landportal.org
	2.2. Soil displacement due to water erosion	Mg/ha/yr	2019	GloSEM: High-resolution global estimates of present and future soil displacement in croplands by water erosion [31].
3. Health and well-being	3.1. Population connected to piped sewers	percent	2020	https://www.who.int
	3.2. Population using safely managed drinking water services	percent	2022	https://www.sdg6data.org/en/indicator/6.1.1
4. Sustainability	4.1. Renewable water resources per capita	${\text{m}}^{3}\;\mathrm{per}\;\mathrm{capita}$	2020–2021	https://www.sdg6data.org/en/indicator/6.4.2
	4.2. Ratio of water abstraction to renewable water	percent	2020–2021	https://data.worldbank.org
				https://www.sdg6data.org/en/indicator/6.4.2
				https://data.worldbank.org

The water productivity of Malaysia, Nigeria, and Turkey is approximately 51, 40, and 16 U S. dollars GDP per cubic meter of water consumed, respectively. These countries have the highest water productivity compared to other countries. The water productivity is less than 8 U S. dollars GDP per cubic meter of water consumed in other countries. Egypt, Malaysia, and Iran have the highest scores in terms of the environmental performance index, which is above 34 %. While the lowest score of this index belongs to Bangladesh and Pakistan, which is less than 25 %. The corruption perception index measures the public perception of corruption. Corruption poses threats to the security of countries, which weakens governments' ability to protect people against these threats. The published corruption perception index by Transparency International indicates that countries fail to stop corruption. Malaysia, Turkey, and Indonesia have a better position in terms of the corruption perception index compared to other countries. The proportion of water key biodiversity areas (KBAs) under protection is one of the indicators of the water environment. The countries with the most extensive water KBAs under protection are Nigeria and Malaysia. The collected values of this indicator have shown that approximately 74 % and 50 % of water KBAs are protected in Nigeria and Malaysia, respectively. The soil displacement due to water erosion, which is another indicator of the water environment, is higher in Indonesia and Malaysia in comparison to other countries. The lowest level of soil displacement belongs to Egypt. The collected values of indicators associated with health and well-being have shown that A high percentage of the population in Turkey, Malaysia, and Egypt are equipped with sewage pipelines. However, Nigeria and Bangladesh are not in a suitable situation in terms of sewage pipeline infrastructure, as less than 10 % of their population are equipped with sewage pipelines. The proportion of the population using safely managed drinking water services is higher than 90 % in Egypt, Turkey, Iran, and Malaysia. In other countries, less than 60 % of their population utilizes these services.

**Fig. 2:**
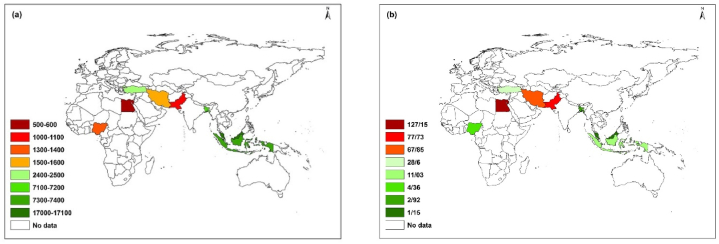
Spatial distribution of sustainability indicators: (a) water availability (cubic meters), (b) water exploitation intensity (%) for selected countries.

The spatial distribution of sustainability indicators for selected countries is shown in Fig. 2. The analysis of sustainability indicators reveals that Malaysia is in the best position with renewable water resources per capita of 17,000 cubic meters, surpassing other countries in the group. Indonesia and Bangladesh also have relatively favorable conditions with renewable water resources per capita of more than 7100 cubic meters, indicating a better situation compared to other countries in terms of this indicator. Egypt, Pakistan, Nigeria, and Iran have the worst position compared to other countries, with renewable water resources per capita of less than 1600 cubic meters (Fig. 2(a)). The ratio of water abstraction to renewable water in Malaysia, Bangladesh, and Nigeria is very low compared to other countries, indicating their better position in this indicator. The water exploitation in these countries is less than 5 %. Egypt, Pakistan, and Iran have very high water exploitation intensity compared to other countries (Fig. 2(b)). Countries can be categorized based on high water availability and low water exploitation, high water availability and high water exploitation, low water availability and low water exploitation, and low water availability and high water exploitation. For example, Egypt, Iran, and Pakistan fall into the group of low water availability (renewable water resources per capita) and high exploitation intensity (ratio of water abstraction to renewable water), while Malaysia falls into the group of high water availability and low water exploitation.

### 2.3 Assessment model

A mathematical programming method to create composite indicators (CIs) is suggested by Zhou et al. (2007). A numerous studies have applied this approach to construct CIs, including de Castro-Pardo et al. (2022) and Hatefi and Torabi (2010). The DEA model is appropriate for constructing CIs when weights of the indicators are not available, or there is no agreement on assigning weight to them [18]. In the present study, this approach has been used to create composite indicators of countries’ WS. It is supposeed that there are n decision-making units whose WS is to be evaluated using m indicators with various measurement units. Model (1) gives the most suitable weights ($w_{ki}^{g}$) for each decision-making unit. ${gI}_{k}$ values are calculated by solving model (1) frequently for each unit according to Equation (1) [32].

(1) $${\text{gI}}_{k}=\max\sum_{i=1}^{m}w_{ki}^{g}I_{\text{ki}}$$

s.t.

$$\sum_{i=1}^{m}w_{ki}^{g}I_{\text{ji}}\leq 1\;j=1,2,\ldots ,n$$

$$w_{ki}^{g}\geq 0\;i=1,2,\ldots ,m$$

Where, $I_{\text{ki}}$ denotes the value of indicator i concerning decision-making unit k; $w_{\text{ki}}$ represents the weight of unit k with relation to indicator i; $I_{\text{ji}}$ is the indicator i for each unit j. Model (1) corresponds to an input-oriented model that includes m outputs and a single dummy input of 1 for all the decision-making units [20]. The model (2) that selects the worst set of weights ($w_{ki}^{b}$) for each unit is as follows [32]:

(2) $${\text{bI}}_{k}=\min\sum_{i=1}^{m}w_{ki}^{b}I_{\text{ki}}$$

s.t.

$$\sum_{i=1}^{m}w_{ki}^{b}I_{\text{ji}}\geq 1\;j=1,2,\ldots ,n$$

$$w_{ki}^{b}\geq 0\;i=1,2,\ldots ,m$$

Model (2) corresponds to an output-oriented model that includes m inputs and a single dummy output of 1 for all the units [20]. Similarly, a set of indicators ${bI}_{1}$, ${bI}_{2}$, …, ${bI}_{n}$ for all units are provided by solving this model. After calculating the ${gI}_{k}$ and ${bI}_{k}$ values for each unit by Equations (1), (2), composite indicators (${CI}_{k}$) of decision-making units are calculated by Equation (3). Here, decision-makers determine the adjustment parameter (γ) [32].

(3) $${\text{CI}}_{k}\left(\gamma \right)=\gamma \;\frac{{\text{gI}}_{k}-{\text{gI}}^{\min}}{{\text{gI}}^{\max}-{\text{gI}}^{\min}}+\left(1-\gamma \right)\;\frac{{\text{bI}}_{k}-{\text{bI}}^{\min}}{{\text{bI}}^{\max}-{\text{bI}}^{\min}}$$

$${\text{gI}}^{\max}=\left\{\max\;{\text{gI}}_{k},k=1,2,3,\ldots ,n\right\}\;\;\;{\text{gI}}^{\min}=\left\{\min\;{\;\text{gI}}_{k},k=1,2,3,\ldots ,n\right\}$$

$${\text{bI}}^{\max}=\left\{\max\;{\;\text{bI}}_{k},k=1,2,3,\ldots ,n\right\}\;\;\;{\text{bI}}^{\min}=\left\{\min\;{\text{bI}}_{k},k=1,2,3,\ldots ,n\right\}$$

0≤γ≤1

Unweighted average (UA) method has been applied to calculate WS indicators (${\text{WS}I}_{\text{ki}}$) of units according to Equation (4) [18].

(4) $${\text{WSI}}_{\text{ki}}=\sum_{i=1}^{m}w_{\text{ki}}I_{\text{ji}}$$

Where, $w_{\text{ki}}$ denotes equal weights concerning indicator i for all the units. In order to measure the WS indicators using the UA model, the values of the indicators have been collected for all decision-making units. The indicators were categorized in the categories of “more is better” (benefit) or “less is better” (cost). After determining the indicator type, All indicators are normalized in order to unify their measurement units. In the present study, the max-min scaling method was used to normalize the original indicators according to Equations (5), (6).

(5) $$X_{norm}^{+}=\frac{X^{+}-X_{\min}}{X_{\max}-X_{\min}}$$

(6) $$X_{norm}^{-}=\frac{X^{-}-X_{\max}}{X_{\min}-X_{\max}}$$

Where, $X_{norm}^{+}$ denotes the normalized amount of the benefit type indicator ($X^{+}$) for each unit; $X_{norm}^{-}$ represents the normalized amount of the cost type indicator ($X^{-}$). Ultimately, the UA method was applied to calculate WS indicators.

## 3 Results and discussion

In the UA method, normalization of different indicators was performed using the max-min method. The normalized results of the indicators for selected countries are shown in Table 2.

**Table 2:** Normalized indicators of WS dimensions for D-8 countries.

	1.1	1.2	1.3	2.1	2.2	3.1	3.2	4.1	4.2
Egypt	0.0731	1.0000	0.2609	0.3863	1.0000	0.8218	1.0000	0.0000	0.0000
Iran	0.0632	0.9194	0.0435	0.4971	0.9718	0.3425	0.9348	0.0617	0.4707
Turkey	0.2984	0.2581	0.5217	0.0565	0.9380	1.0000	0.9751	0.1180	0.7822
Indonesia	0.0589	0.4113	0.4348	0.5289	0.0000	0.0289	0.0185	0.4107	0.9216
Nigeria	0.7736	0.4194	0.0000	1.0000	0.6789	0.0000	0.0000	0.0473	0.9745
Bangladesh	0.1157	0.0000	0.0435	0.0000	0.6423	0.0038	0.4317	0.4010	0.9859
Pakistan	0.0000	0.1210	0.1304	0.4868	0.8435	0.2296	0.3098	0.0315	0.3922
Malaysia	1.0000	0.9597	1.0000	0.6781	0.4160	0.8795	0.9308	1.0000	1.0000

It should be noted that the normalized indicators, which are close to zero for some countries, do not imply zero or low values for those indicators. Instead, they emphasize the distance between the values of those indicators for a specific country compared to countries with the best scores in those indicators. After normalizing the indicators, the UA method was used according to model (4) to create composite indicators for the overall dimensions of WS. The normalized values of the composite indicators for the overall dimensions using the UA method, as well as the ranking of selected countries, are presented in Table 3.

**Table 3:** The composite indicators of WS related to the overall dimensions using the DEA and UA models.

Country	DEA			Rank DEA	UA	Rank UA
	Model 1	Model 2	Model 3			
Egypt	1	1	0.5000	2	0.371	3
Iran	0.978	1	0.4679	5	0.340	4
Turkey	1	1	0.5000	2	0.482	2
Indonesia	0.805	1	0.2157	6	0.012	7
Nigeria	1	1	0.5000	2	0.234	5
Bangladesh	0.657	1	0.0000	8	0.025	6
Pakistan	0.71	1	0.0773	7	0.000	8
Malaysia	1	1.284	1.0000	1	1.000	1

For assessing the WS using the DEA model, the primary indicators were utilized. The DEA model, similar to models (1) and (2), was executed using the Win4Deap2 software. Subsequently, the composite indicator of WS was obtained for all countries using the outputs of model (1), model (2), and γ = 0.5 in model (3). Finally, all selected countries were ranked based on the derived values of the composite indicator for the overall dimensions of WS. The normalized values of the composite indicator for the overall dimensions of WS using the DEA model, as well as the ranking of selected countries, are presented in Table 3.

The analysis of the results obtained from the DEA model, considering all dimensions of WS, indicated that Malaysia is in a better position in terms of WS compared to other countries, while Bangladesh and Pakistan are in the lowest positions. Comparing the outcomes from the DEA and UA methods, we can see that only Malaysia (1st) and Turkey (2nd) share the same positions. The results obtained from the UA method show that Pakistan and Indonesia are in the lowest positions. They have the lowest composite indicator of WS compared to other countries.

WS involves numerous dimensions, some of which are influenced by human activities and environmental factors. Lack of management or mismanagement in these dimensions has reduced the overall composite indicator of WS for countries. These issues can significantly affect country rankings, which have caused incorrect rankings. Therefore, if all dimensions of WS are considered together, it is possible that the ranking does not accurately reflect the WS status of countries. With regard to certain dimensions of WS, such as sustainability, which have significant effects on determining the WS status of countries, it is recommended to have a basis of comparison based on the dimensions of WS for a detailed examination and accurate ranking of countries. By doing this, the status of countries can be independently compared in each dimension. Finally, appropriate solutions and actions should be tailored to the specific status of each country in order to improve WS. Another point is that special attention should be given to the sustainability dimension.

Composite indicators of WS were developed for selected countries in each dimension using the DEA model, according to models (1) to (3). Countries were then ranked based on the normalized values of the composite indicator. The normalized values of the composite indicator related to the dimension of functions and responsibilities of societies using the DEA model, as well as the ranking of selected countries, are presented in Table 4.

**Table 4:** The composite indicators of WS related to the dimension of functions and responsibilities of societies using the DEA model.

Country	DEA			Rank DEA
	Model 1	Model 2	Model 3	
Egypt	1	1.177	0.6718	2
Iran	0.972	1	0.4594	3
Turkey	0.766	1.139	0.2958	5
Indonesia	0.803	1.174	0.3834	4
Nigeria	0.808	1	0.2217	6
Bangladesh	0.655	1	0.0000	8
Pakistan	0.698	1	0.0623	7
Malaysia	1	1.515	1.0000	1

The results of WS in the dimension of functions and responsibilities of societies indicated that Malaysia, Egypt, Iran, Indonesia, Turkey, Nigeria, Pakistan, and Bangladesh hold the first to eighth rankings, respectively. Among the selected countries, Malaysia has a higher WS in this dimension compared to other countries. The lowest composite indicator in this dimension belongs to Bangladesh and Pakistan, indicating their lower scores in comparison to other countries. The normalized values of the composite indicator for the dimension of the water environment using the DEA model, as well as the ranking of selected countries, are given in Table 5.

**Table 5:** The composite indicators of WS related to the dimension of the water environment using the DEA model.

Country	DEA			Rank DEA
	Model 1	Model 2	Model 3	
Egypt	1	64.963	1.0000	1
Iran	0.595	11.35	0.3751	3
Turkey	0.107	5.443	0.0810	7
Indonesia	0.529	1	0.2607	6
Nigeria	1	2.32	0.5103	2
Bangladesh	0.016	1	0.0000	8
Pakistan	0.503	2.829	0.2618	5
Malaysia	0.678	1.435	0.3398	4

The results of WS in the dimension of water environment depicted that Egypt and Nigeria have higher scores in comparison with other countries. By comparing the indicators related to the environmental dimension of the selected countries, it was found that the lowest soil erosion and the highest proportion of water KBAs under protection were in Egypt and Nigeria, respectively. These factors have caused these countries to obtain higher scores in the dimension of water environment. Bangladesh and Turkey have lower scores than other countries in this dimension. By comparing the indicators related to the environmental dimension of the selected countries, it was revealed that the lowest proportion of water KBAs under protection was in Bangladesh and Turkey, which caused these countries to earn lower scores.

The normalized values of the composite indicator for the dimension of health and well-being using the DEA model, as well as the ranking of selected countries, are provided in Table 6.

**Table 6:** The composite indicators of WS related to the dimension of health and well-being using the DEA model.

Country	DEA			Rank DEA
	Model 1	Model 2	Model 3	
Egypt	1	3.408	1.0000	1
Iran	0.954	3.251	0.9349	4
Turkey	1	3.348	0.9875	2
Indonesia	0.306	1.045	0.0185	7
Nigeria	0.293	1	0.0000	8
Bangladesh	0.598	1.033	0.2226	6
Pakistan	0.512	1.746	0.3098	5
Malaysia	0.96	3.242	0.9372	3

The analysis of results in this section indicated that Egypt, Turkey, Malaysia, and Iran have better conditions and higher scores in the dimension of health and well-being compared to other countries. On the other hand, Nigeria and Indonesia have lower scores in this dimension compared to other countries. The normalized values of the composite indicator for the dimension of sustainability using the DEA model, as well as the ranking of selected countries, are tabulated in Table 7.

**Table 7:** The composite indicators of WS related to the dimension of sustainability using the DEA model.

Country	DEA			Rank DEA
	Model 1	Model 2	Model 3	
Egypt	0.031	1	0.0000	8
Iran	0.091	1.875	0.0448	6
Turkey	0.145	4.449	0.1135	5
Indonesia	0.429	11.538	0.3725	3
Nigeria	0.264	2.493	0.1439	4
Bangladesh	0.419	13.646	0.4007	2
Pakistan	0.061	1.637	0.0256	7
Malaysia	1	32.535	1.0000	1

The results of WS in terms of sustainability indicated that Malaysia, Bangladesh, Indonesia, Nigeria, and Turkey have better positions compared to other countries. These countries have assigned themselves first to fifth ranks, respectively. The intensity of water exploitation in all of these countries was lower than 40 %, which affects the creation of WS in the sustainability dimension. In addition, each person in Malaysia, Bangladesh, and Indonesia has access to more than 7000 cubic meters of water, which increases the WS of countries in terms of sustainability. The high water exploitation and low water availability aggravate the insecure situation in the sustainability dimension in Iran, Pakistan, and Egypt.

**Fig. 3:**
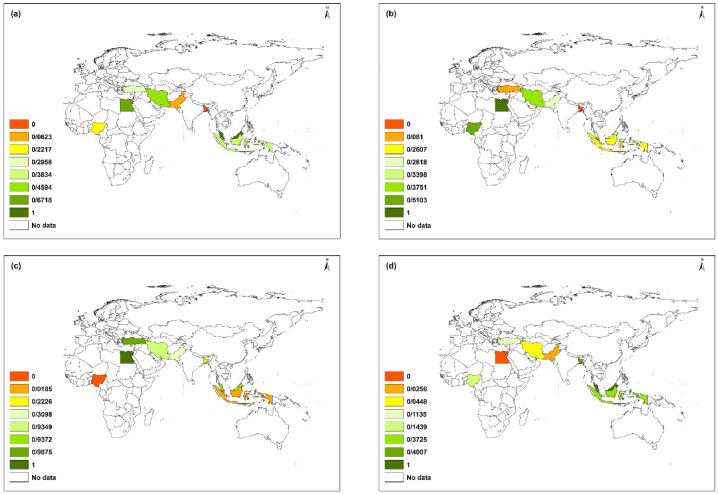
Spatial distribution of WS in different dimensions: (a) functions and responsibilities of societies; (b) water environment; (c) health and well-being; (d) sustainability for the selected countries.

The current situation of WS can be presented at different spatial levels. The spatial distribution of WS in different dimensions for selected countries is shown in Fig. 3.

The ranking outcomes of the composite indicators in various dimensions for selected countries utilizing the DEA model are presented in Table 8.

**Table 8:** Ranking of selected countries based on the composite indicators of WS dimensions using the DEA model.

Country	Rank DEA			
	Dimension 1 (functions and responsibility)	Dimension 2 (water environment)	Dimension 3 (health and well-being)	Dimension 4 (sustainability)
Egypt	2	1	1	8
Iran	3	3	4	6
Turkey	5	7	2	5
Indonesia	4	6	7	3
Nigeria	6	2	8	4
Bangladesh	8	8	6	2
Pakistan	7	5	5	7
Malaysia	1	4	3	1

It is worth noting that some countries may receive higher scores in one dimension of WS while receiving lower scores in other dimensions. The results extracted from the DEA model indicated that Malaysia had a high level of WS in the dimensions of sustainability and functions and responsibilities of societies compared to other countries, But it ranked third and fourth in the dimensions of health and well-being and water environment, respectively. The ranking of selected countries in terms of WS in the sustainability dimension was as follows: Malaysia, Bangladesh, Indonesia, Nigeria, Turkey, Iran, Pakistan, and Egypt, respectively. Although some countries like Bangladesh had high WS in the sustainability dimension, they had lower WS in the dimensions of water environment and functions and responsibilities of societies compared to other countries. Despite having the lowest score in terms of the sustainability dimension for some countries like Egypt, they had the highest score in the dimension of health and well-being in comparison with other countries in the group.

In other studies, all dimensions have been considered together. For example, de Castro-Pardo et al. (2022) have applied the DEA approach to construct CIs in European countries. In this study, European countries are ranked in terms of the total dimensions. Park et al. (2020) have selected 28 Asian countries [33]. The results of the WS status of the common countries are consistent with the results of our study, considering all dimensions. In the present investigation, WS has been evaluated separately in each dimension. By doing this, the status of countries can be independently compared in each dimension. The current investigation has helped to identify insecure countries in terms of each dimension. Therefore, knowledge of the weak dimensions is a good guide for suggestions and necessary actions to improve WS. The outcomes demonstrated that Iran, Pakistan, and Egypt have unsustainable situations. 5.5 % of the global population lives in these countries, so managers should look for solutions to promote sustainable water consumption. Factors that may improve their situation in different dimensions include advanced irrigation techniques, educating farmers on water-smart agricultural practices, water recycling and reuse, improving water infrastructure and management, and public education. The countries must consider appropriate actions based on their situation.

## 4 Conclusions

In the present study, the WS status of D-8 countries was examined. To achieve this goal, the DEA model was used to create composite indicators of the WS dimensions of the countries. Selected countries were then ranked based on the normalized values of the composite indicators. It is worth noting that the basis of the work is only comparing the countries. When some countries have higher scores than other countries, it does not imply that they have a good situation. The outcomes obtained from the DEA model demonstrated that Malaysia, Bangladesh, Indonesia, Nigeria, and Turkey have better situations in terms of sustainability than the other countries. The water exploitation intensity in these regions is less than 40 %. Moreover, the level of water availability in Malaysia, Bangladesh, and Indonesia exceeds 7000 cubic meters per person. The low water exploitation and high water availability in these regions have significant effects on achieving WS in the sustainability dimension. The problems such as very high water exploitation and low water availability in Iran, Pakistan, and Egypt have aggravated their water insecurity in the sustainability dimension. While Bangladesh has the highest score in the sustainability dimension after Malaysia, it has the lowest score in the dimensions of water environment and functions and responsibilities of societies compared to other countries. It is noteworthy that certain countries may have higher levels of WS in a particular dimension while simultaneously having lower levels of WS in another dimension. As a result, it is suggested that the dimensions of WS can be used as the basis for comparison. This recommendation says that countries' WS should be assessed and discussed separately in each security dimension. In this way, it is possible to compare the situation of countries independently in each security dimension. Ultimately, appropriate solutions and actions should be provided to improve the WS of countries based on the state of countries in each security dimension. It should be noted that the sustainability dimension reflects the level of water availability and water exploitation intensity. Since the sustainability dimension has a significant and influential role in determining the WS status of countries, it is recommended to pay special attention to the sustainability dimension. Another important point is that results will differ with the addition of new indicators or new countries. It should be noted that one of the limitations of this study is the lack of updated data, however, we are forced to use the available data.

## CRediT authorship contribution statement

**Azam Bahramifard:** Writing – review & editing, Writing – original draft, Software, Methodology, Investigation, Data curation, Conceptualization. **Mansour Zibaei:** Writing – review & editing, Supervision, Methodology, Investigation, Conceptualization.

## Data availability statement

Data will be made available on request.

## Declaration of competing interest

The authors declare that they have no known competing financial interests or personal relationships that could have appeared to influence the work reported in this paper.

## References

[1] Biswas A.K., Tortajada C. (2016).

[2] Salim N., Anziani-Vente M., Madsen D., Lim K., Makarigakis A.K., Sohn O., Lee B. (2019). Water Security and the Sustainable Development Goals.

[3] Van Beek E., Arriens W.L. (2014).

[4] Veettil A.V., Mishra A. (2020). Water security assessment for the contiguous United States using water footprint concepts. Geophys. Res. Lett..

[5] Escap U. (2013).

[6] Tortajada C., Fernandez V. (2018). Towards global water security: a departure from the status quo?. Global Water Security: Lessons Learnt and Long-Term Implications.

[7] Hoekstra A.Y., Buurman J., Van Ginkel K.C. (2018). Urban water security: a review. Environ. Res. Lett..

[8] Robinne F.-N., Bladon K.D., Miller C., Parisien M.-A., Mathieu J., Flannigan M.D. (2018). A spatial evaluation of global wildfire-water risks to human and natural systems. Sci. Total Environ..

[9] Su Y., Gao W., Guan D. (2019). Integrated assessment and scenarios simulation of water security system in Japan. Sci. Total Environ..

[10] Bakker K. (2012). Water security: research challenges and opportunities. Science.

[11] Cook C., Bakker K. (2012). Water security: debating an emerging paradigm. Global Environ. Change.

[12] Li X., Su X., Wei Y. (2019). Multistage integrated water security assessment in a typical region of Northwestern China. J. Clean. Prod..

[13] Grey D., Sadoff C.W. (2007). Sink or swim? Water security for growth and development. Water Pol..

[14] Zeitoun M. (2011). The global web of national water security. Global Policy.

[15] D'Ambrosio E., Ricci G.F., Gentile F., De Girolamo A.M. (2020). Using water footprint concepts for water security assessment of a basin under anthropogenic pressures. Sci. Total Environ..

[16] Jensen O., Wu H. (2018). Urban water security indicators: development and pilot. Environ. Sci. Pol..

[17] Mishra B.K., Kumar P., Saraswat C., Chakraborty S., Gautam A. (2021). Water security in a changing environment: concept, challenges and solutions. Water.

[18] de Castro-Pardo M., Fernández Martínez P., Pérez Zabaleta A. (2022). An initial assessment of water security in Europe using a DEA approach. Sustainable Technology and Entrepreneurship.

[19] Pérez Zabaleta A., Fernández P., Prados-Castillo J.F., de Castro-Pardo M. (2022). Constructing fuzzy composite indicators to support water policy entrepreneurship. Sustainable Technology and Entrepreneurship.

[20] Hatefi S.M., Torabi S.A. (2010). A common weight MCDA–DEA approach to construct composite indicators. Ecol. Econ..

[21] Bin O., Shuyan F., Yu W., Liping W. (2012). The comprehensive evaluation of rural drinking water security in Yunnan Province. Procedia Earth and Planetary Science.

[22] Chen J., Xia L., Wang H. (2011). Modeling Risk Management for Resources and Environment in China.

[23] Dai J., Qi J., Chi J., Chen S., Yang J., Ju L., Chen B. (2010). Integrated water resource security evaluation of Beijing based on GRA and TOPSIS. Front. Earth Sci. China.

[24] Li B., Wu Q., Zhang W., Liu Z. (2020). Water resources security evaluation model based on grey relational analysis and analytic network process: a case study of Guizhou Province. J. Water Proc. Eng..

[25] Liu L., Tang D., Chen T. (2012). 2012 IEEE Fifth International Conference on Advanced Computational Intelligence (ICACI).

[26] Lu S., Wang J., Bao H. (2016). Study on urban water security evaluation based on the vague set similarity model. Energy Proc..

[27] Qadeer T., Li Z. (2018).

[28] Tu Y., Chen K., Wang H., Li Z. (2020). Regional water resources security evaluation based on a hybrid fuzzy BWM-TOPSIS method. Int. J. Environ. Res. Publ. Health.

[29] Yang Y., Hu H., Xue H. (2022). Water security comprehensive evaluation model based on comprehensive weight and improved matter-element. Rev. Int. Contam. Ambient..

[30] Marttunen M., Mustajoki J., Sojamo S., Ahopelto L., Keskinen M. (2019). A framework for assessing water security and the water–energy–food nexus—the case of Finland. Sustainability.

[31] Borrelli P., Ballabio C., Yang J.E., Robinson D.A., Panagos P. (2022). GloSEM: high-resolution global estimates of present and future soil displacement in croplands by water erosion. Sci. Data.

[32] Zhou P., Ang B.W., Poh K.L. (2007). A mathematical programming approach to constructing composite indicators. Ecol. Econ..

[33] Park S.-Y., Lee S., Lee H.-J., Lee J.-H. (2020). Water security assessment of Asian countries for sustainable water management. J. Korea Water Resour. Assoc..

